# 

**Published:** 1868-06

**Authors:** 


### CONTENTS:

### ORIGINAL COMMUNICATIONS.

ARTICLE I.—Treatment of Fracture of Lower Jaw by Interdental Splints. By Thomas Brian Gunning, D. D. S. .......... 53
ARTICLE II.—Diagnosis in Fang Operations. By Benj. F. Cosby, D. D. S. .......... 66
ARTICLE III.—Case of Fracture of Upper and Lower Jaw. By T. O. Oliver .......... 68
ARTICLE IV.—Nitrous Oxide Gas. By R. N. Hudson, D. D. S. .......... 69

### CORRESPONDENCE.

ARTICLE V.—Proceedings of the Association of the Colleges of Dentistry. By J. Taft, D.D.S. 71

ARTICLE VI.—Illinois State Dental Society .......... 76

ARTICLE VII.—Letter from Paris. By D'Oyley Evans .......... 84

### SELECTED ARTICLES.

ARTICLE VIII.—Foreign Bodies in the Ear. By Henry L. Shaw, M. D. .......... 86

ARTICLE IX.—Colorless Tincture of Iodine. By Hubert Primm, Ph. D. .......... 93

### MONTHLY SUMMARY.

A Monster Tumor .......... 95

Alopecia, Causes of .......... 96

"With Brains, Sir." .......... 98

Keratitis .......... 98

External use of Arnica .......... 99

### BIBLIOGRAPHICAL NOTICES.

Taft's Operative Dentistry.—Second Edition .......... 99

### EDITORIAL DEPARTMENT.

The American Dental Association .......... 102

Filling Pulp Cavities .......... 103

Treatment of Dental Pulp .......... 103

Time of Meeting of American Dental Association .......... 104

A Method for removing Amalgam Fillings .......... 104

The Rubber Question .......... 105

